# Therapeutic Effects of Anti-CD115 Monoclonal Antibody in Mouse Cancer Models through Dual Inhibition of Tumor-Associated Macrophages and Osteoclasts

**DOI:** 10.1371/journal.pone.0073310

**Published:** 2013-09-03

**Authors:** Laetitia Fend, Nathalie Accart, Jacqueline Kintz, Sandrine Cochin, Carine Reymann, Fabrice Le Pogam, Jean-Baptiste Marchand, Thierry Menguy, Philippe Slos, Ronald Rooke, Sylvie Fournel, Jean-Yves Bonnefoy, Xavier Préville, Hélène Haegel

**Affiliations:** 1 Transgene, Illkirch-Graffenstaden, France; 2 UMR 7199 CNRS-Université de Strasbourg, Faculté de Pharmacie, Illkirch-Graffenstaden, France; University of Lyon, France

## Abstract

Tumor progression is promoted by Tumor-Associated Macrophages (TAMs) and metastasis-induced bone destruction by osteoclasts. Both myeloid cell types depend on the CD115-CSF-1 pathway for their differentiation and function. We used 3 different mouse cancer models to study the effects of targeting cancer host myeloid cells with a monoclonal antibody (mAb) capable of blocking CSF-1 binding to murine CD115. In mice bearing sub-cutaneous EL4 tumors, which are CD115-negative, the anti-CD115 mAb depleted F4/80^+^ CD163^+^ M2-type TAMs and reduced tumor growth, resulting in prolonged survival. In the MMTV-PyMT mouse model, the spontaneous appearance of palpable mammary tumors was delayed when the anti-CD115 mAb was administered before malignant transition and tumors became palpable only after termination of the immunotherapy. When administered to mice already bearing established PyMT tumors, anti-CD115 treatment prolonged their survival and potentiated the effect of chemotherapy with Paclitaxel. As shown by immunohistochemistry, this therapeutic effect correlated with the depletion of F4/80^+^CD163^+^ M2-polarized TAMs. In a breast cancer model of bone metastasis, the anti-CD115 mAb potently blocked the differentiation of osteoclasts and their bone destruction activity. This resulted in the inhibition of cancer-induced weight loss. CD115 thus represents a promising target for cancer immunotherapy, since a specific blocking antibody may not only inhibit the growth of a primary tumor through TAM depletion, but also metastasis-induced bone destruction through osteoclast inhibition.

## Introduction

Macrophages and osteoclasts are myeloid cell types known to contribute to cancer progression at various stages of the disease [1]–[5]. Their differentiation and function are regulated by CD115 (M-CSFR, CSF-1R, c-fms), encoded by the *c-fms* proto-oncogene and belonging to the class III receptor tyrosine kinase family [6]. CD115 is the sole cell-surface receptor identified to date for colony-stimulating factor-1 (CSF-1), a major cytokine regulating the differentiation, proliferation and migration of myeloid lineage cells [7]. Interleukin-34 (IL-34) has more recently been identified as another CD115 ligand with comparable biological effects [8]. While the regulation and function of IL-34 during cancer progression remain to be investigated, experimental and clinical evidence have largely documented the central role of CSF-1 in tumor development and metastasis.

In humans, CD115 and CSF-1 overexpression are frequent in a wide variety of epithelial tumors (breast, prostate, endometrial, cervical, ovarian cancers) and have been correlated with more aggressive diseases and poor prognosis [9]–[13]. In breast tumors, CD115 was found to be expressed both by tumor cells and by infiltrating macrophages [14]. It was suggested by S. Scholl *et al*. [15] that CSF-1 might not only act as an autocrine growth factor for tumor cells, but also recruit macrophages to the tumor site promoting tumor progression. In the MMTV-PyMT (PyMT) mouse model of spontaneous breast cancer, CSF-1 produced by tumor cells was indeed shown to be an important chemoattractant for macrophages and to enhance their infiltration into the primary tumor [16]. Tumor-associated macrophages (TAMs) regulate the angiogenic switch through induction of angiogenic factors such as vascular endothelial growth factor and produce matrix-metalloproteases, which can regulate angiogenesis and facilitate metastasis [17], [18]. Metastatic cells require CD115-positive macrophages for extravasation and growth into their metastatic site [5]. *In vivo* invasion assays have shown that TAMs co-migrate with breast tumor cells and contribute to tumor cell invasion through a paracrine loop involving epidermal growth factor, produced by macrophages, and CSF-1 produced by cancer cells [19]–[21]. In addition, CSF-1 has been shown to polarize macrophages towards an alternatively-activated, trophic or M2-type, endowed with immunosuppressive activity and characterized notably by CD163 expression [22]–[28]. High numbers of TAMs, which can constitute the most abundant immunosuppressive cell population in the tumor microenvironment, have been correlated with bad prognosis in many cancers including breast [1], [2], [4], [23]. Because of their pleiotropic roles in tumor progression, TAMs represent an important target for cancer therapy [29].

CSF-1 overexpression by bone metastases may also contribute to the differentiation of osteoclasts, leading to bone lesions and pain in cancer patients. Osteoclasts, like macrophages, are dependent on the CD115/CSF-1 pathway for their differentiation [30]. CSF-1 notably induces RANK expression by osteoclast precursors [31]–[33]. Recent results indicate that CSF-1 is a potent stimulator of mature osteoclast bone-resorbing activity, in addition to RANK/RANKL [32]. Both cell-surface and secreted CSF-1 produced by tumor cells metastatic to bone can contribute to induce osteoclast formation [3].

We have studied the effects of targeting CD115 in 3 different mouse cancer models characterized by the infiltration of M2-polarized TAMs in the primary tumors or by the formation of osteolytic bone metastases. To this purpose, we used a monoclonal antibody (mAb), AFS98, known to block CSF-1 binding to murine CD115-expressing cells and to inhibit CSF-1-dependent colony formation by mouse bone marrow precursors [34]. This mAb has been used by other authors to deplete myeloid cell subsets in various experimental mouse models [19], [29], [35], [36].

Our results highlight the potential of cancer immunotherapy with an anti-CD115 mAb, which can inhibit tumor growth by depleting TAMs, synergize with chemotherapy and prevent bone destruction by osteoclasts.

## Materials and Methods

### Antibodies

The hybridoma AFS98 secreting rat anti-mouse CD115 IgG_2a_ was kindly provided by Pr S. Nishikawa (RIKEN Center for Developmental Biology, Japan). The mAb was produced by culture of the hybridoma in complete RPMI medium containing 5% FCS and purified on protein G (Millipore) and diafiltration on Pellicon 3 30 kDA (Millipore). Rat IgG used as negative control was from Rockland/Tebu-bio. In some instances, mAb AFS98 and isotype control rat IgG_2a_ were purchased from eBiosciences.

### Cell Lines

The EL4 murine lymphoma cell line (ATCC TIB-39) was cultured in complete DMEM medium (Sigma-Aldrich) with 10% FCS (PAA Laboratories) with 40 µg/mL gentamycin (Schering-Plough) and 2 mM glutamine (Sigma). MDA-MB231SA-GFP6 human breast cancer cells used in the metastasis-induced osteolysis model were obtained by Pharmatest (Turku, Finland) from Dr Theresa Guise (Indiana University, Indianapolis, USA) [37], [38] and transfected with pTurboGFP-N vector (Evrogen).

### Immunofluorescence

Mouse formalin-fixed paraffin-embedded tissue sections were deparaffinized and rehydrated, and epitopes were retrieved by boiling in a 10 mM Citrate Buffer pH 6. EL-4 cryosections were rehydrated and fixed in acetone. Sections were saturated with 3% H2O2 and 10% goat serum to eliminate endogenous peroxidase activity and non-specific staining, respectively, before incubation for 90 min at room temperature with antibodies against F4/80 (BM8, ref. MF48000, Caltag) or CD163 (M-96, ref. sc-33560, Santa Cruz). Envision system (HRP-conjugated polymer coupled to secondary antibodies, ref. K4003, Dako) and TSA-Cy3 (ref. SAT704A, Perkin Elmer) was used for signal amplification. Finally sections were counterstained with 0.5 µg/mL DAPI (Hoechst 33258, B-2883, Sigma) and mounted in Mowiol® (Calbiochem). Isotype-matched control antibodies were used as negative controls. Images were acquired with an optical microscope 90i (Nikon) equipped with 40x objective and epifluorescence (scale bar = 50 µm). Signal was quantified on section scans (Nanozoomer, Hamamatsu) using the ImageJ (http://rsb.info.nih.gov/ij) free software. To this aim, images were split into 3 channels (red, green and blue). The blue channel allows quantifying the blue pixels defining the tumor section surface, while the red channel measures the red pixels corresponding to the immunostained surface. The percentages of positive cells are calculated as (red pixels/blue pixels) x100.

### Preclinical Mouse Models

Experiments performed in the EL-4 and PyMT cancer models were performed in full compliance with the CEE directive 2010/63 of September 22^d^, 2010 relating to the protection of animals used for experimental or other scientific purposes and in compliance with the French law (décret n° 2013–118 of February 1^st^, 2013) and approved by the Comité National de Réflexion Ethique sur l’Experimentation Animale (CNREEA, Ethical Committee TG number 93). The experiments in the breast cancer bone metastasis model were approved by the Finnish National Committee for Animal Experiments (license number STH667A).

### EL4 Tumor Model

Five-week old C57BL/6 mice (Charles River Laboratories) were housed in a specific-pathogen free animal facility and acclimated for one week before the experiments. At day 0, 10^4^ EL-4 cells were injected subcutaneously into the right flanks of mice. Ten days after tumor implantation, mAbs diluted in PBS were dosed IP at 10, 25 or 50 mg/kg to groups of 21 mice. Injections were performed 3 times per week for 3 weeks. Tumor volumes were monitored twice a week by caliper measurements of each dimension and calculated using the following formula: V = 4/3 π (length/2)(width/2)(depth/2). Three mice per group bearing EL4 tumors were sacrificed at days 20 and 27, when tumors were sampled for macrophages detection by immunofluorescence. Mice were euthanized by cervical dislocation when tumor volumes reached 3000 mm^3^ or when animals showed distress, to avoid unnecessary suffering. Statistics on tumor volumes (n = 15 mice/group) were performed using the Kruskal-Wallis test followed by Mann-Whitney’s test for pairwise comparisons**.** Log-rank test was used for the statistical analysis of mouse survival (n = 15).

### PyMT Tumor Model

MMTV-PyMT (PyMT) transgenic mice were obtained from the Mayo Foundation for Medical Education and Research (Rochester, Minnesota) and housed in a specific-pathogen free animal facility. The strain was maintained by backcrossing on the C57BL/6 background. When PyMT mice reached 10 weeks of age (early treatment), 10 mice per group were administered with mAbs through the IP route, 3 times per week for 4 weeks for studying tumor growth and mouse survival. Late treatment was performed similarly but starting from week 16. MAbs were given at the dose of 50 mg/kg, diluted in PBS. Tumor volumes were monitored every 7 days by caliper measurements of 3 dimensions and calculated using the following formula: V = 4/3 π (length/2)(width/2)(depth/2). To avoid unnecessary suffering, mice were euthanized by cervical dislocation when total tumor volumes, i.e. the sum of all measurable tumors, were above 2000 mm^3^. Statistics on tumor volumes were performed using the Kruskal-Wallis test followed by Mann-Whitney’s test for pairwise comparisons**.** Statistics on survival were performed using the Log-rank test. In repeat experiments performed on 3 mice per group, the animals were sacrificed by cervical dislocation one week after the termination of the treatment. Three to 5 tumors per mice were sampled for immunofluorescence and histopathology.

### Histopathology

The progression of primary tumors was analyzed on tumor sections from 4 to 25 week-old PyMT mice. Briefly, mammary glands were fixed in 10% formalin at 4°C and then dehydrated. Tissues were paraffin-embedded, sectioned, and stained with hematoxylin and eosin (H&E). Tumor staging was performed based on the four-stage classification previously described for PyMT mice [39].

### MDA-MB231 Breast Cancer Metastasis-induced Osteolysis Model

Five-week old female athymic nude mice (Harlan) anesthetized with Xylazin (4–5 mg/ml) and Ketamine (75–92 mg/ml) were inoculated with MDA-MB231SA-GFP6 cells (10^5^ cells in 0.1 ml of PBS) by the intracardiac route. Allocation to groups of 15 mice was performed by randomization procedure based on body weight. Treatment was started on the day of intracardiac inoculation, at day 0. Fifty mg/kg of mAb AFS98 or control rat IgG (Rockland, Tebu-bio) were administered IP 3 times per week, while zoledronic acid (Zometa, Novartis) was injected SC at 0.1 mg/kg on days 0 and 14. Analgesia (buprenorphine 0.1 mg/kg, SC twice a day) was used for the last 5 days of the study. Blood samples for analyzing tartrate-resistant acid phosphatase isoform 5b (TRACP 5b) were collected before the beginning of administration and at days 17 and 24. Serum TRACP 5b was analyzed using MouseTRAP™ ELISA kit (IDS Ltd). Statistical analysis of the results was performed using one-way ANOVA after logarithmic transformation of the data, followed by Dunnett’s test for pairwise comparisons. Osteolysis was measured by radiographic analysis of the animals under anesthesia prior to sacrifice, using the Faxitron Specimen Radiographic System MX-20 D12 (Faxitron Corp.) and Faxitron Dicom 3.0–software. At least two radiographs (both hind limbs and thorax) per animal were taken each time. The lesion number and area in hind limbs was determined from the images with MetaMorph image analysis software. Statistical analysis was performed using Kruskal-Wallis test followed by Mann-Whitney’s test for pairwise comparisons. Mice were sacrificed at day 24 by cervical dislocation under anesthesia. Relative body weight at sacrifice was calculated as a percentage relative to day 0. Statistical analysis was performed using one-way ANOVA followed by Dunnett’s test for pairwise comparisons. Four 4 µm thick mid-sagittal sections were obtained from both hind limbs of each animal. Trabecular bone areas and tumor area were measured from Masson-Goldner trichrome-stained sections and osteoclasts in tumor-bone interface were calculated from TRACP-stained sections. The number of osteoclasts was divided by the length of tumor-bone interface. Statistical analysis was performed using one-way ANOVA followed by Dunnett’s test for pairwise comparisons.

## Results

### Anti-CD115 mAb Depletes TAMs and has Therapeutic Effect in a Solid Tumor Model

Preliminary experiments were conducted to assess the mode of action of mAb AFS98. Competition ELISA were performed, in which soluble murine CD115-Fc was co-incubated with immobilized murine or human CSF-1 in the presence of increasing AFS98 concentrations (**Figure S1**). AFS98 blocked the binding of CSF-1 from both species to mouse CD115, showing that its inhibitory effect on the receptor function most probably relied on competition with the ligand.

**Figure 1:**
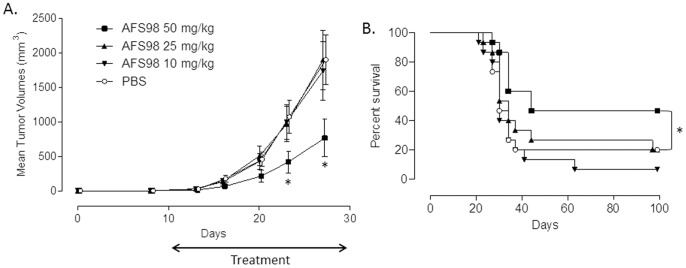
Therapeutic effects of anti-mouse CD115 mAb in the EL4 solid tumor model. Tumor-bearing mice were treated with 10, 25 or 50 mg/kg of mAb AFS98 or PBS, IP, starting from day 7 after tumor implantation. **A**. Tumor volumes are shown as means ± SEM. * Mann-Whitney’s test p<0.05 compared with the tumor volumes of PBS-treated mice (n = 15). **B**. Percentages of surviving mice in each group. * Log-rank test p<0.05 compared with the PBS-treated mouse group (n = 15).

**Figure 2:**
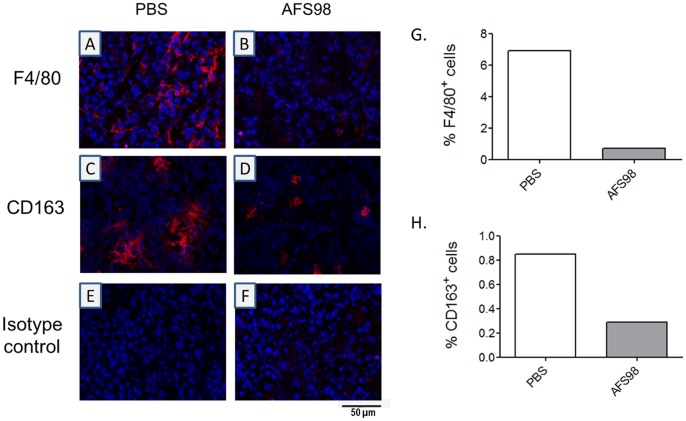
Depletion of M2-polarized TAMs by anti-mouse CD115 mAb in EL4 solid tumors. EL-4 tumors sampled at day 20 from mice treated with either PBS (A, C, E) or 50 mg/kg mAb AFS98 (B, D, F) were analyzed by IF with anti-F4/80 mAb (A, B), anti-CD163 mAb (C, D) or irrelevant isotype control (E, F). Blue : DNA; Red : F4/80^+^ macrophages (A, B) or CD163^+^ M2-type macrophages (C, D). Sections shown were obtained from one tumor representative of 3 analyzed per group. F4/80 (G) and CD163 (H) staining were quantified in the tumors shown in A-D.

The effect of targeting CD115 with mAb AFS98 on tumor growth was first studied in a cancer model using EL4 thymoma cells implanted subcutaneously in the flanks of C57BL/6 mice. EL4 cells are CD115-negative *in vitro* as determined by IC/FC with mAb AFS98 (data not shown). Tumor growth was retarded in mice treated for 3 weeks with the anti-CD115 mAb starting ten days after implantation, compared to PBS-treated mice (**Figure 1A**). Only the maximal tested dose of 50 mg/kg produced a statistically significant reduction in tumor sizes (p = 0.0245 and p = 0.0166 (n = 15) at days 23 and 27, respectively), while there was no effect with 10 or 25 mg/kg. Consequently, survival was significantly prolonged (p = 0.0368) only in mice treated with 50 mg/kg AFS98 (**Figure 1B**). F4/80 staining by IHC showed that the macrophage population was strongly reduced in tumors sampled during AFS98 treatment, both at days 20 (**Figure 2A, B**) and 27 (data not shown). Expression of the M2-type macrophage marker CD163 was also considerably decreased in EL4 tumors from mice treated with 50 mg/kg AFS98 (**Figure 2C, D**). These results suggest that the therapeutic effect of the anti-CD115 mAb in this tumor model may be a consequence of M2-type TAM depletion.

Because CSF-1 is normally cleared from the circulation by CD115-mediated endocytosis [40] and since AFS98 prevents CSF-1/CD115 binding, we titrated the levels of circulating CSF-1 after only one mAb injection. As soon as one day post-administration, AFS98 at all doses tested dramatically increased CSF-1 serum concentrations (**Figure S2**). While serum CSF-1 was hardly detectable by ELISA in buffer-treated mice, its concentration reached 2000 pg/ml one day after AFS98 injection. The importance and duration of CSF-1 accumulation in the serum was correlated with the doses of AFS98 administered. At 25 or 50 mg/kg, AFS98 had an even more drastic effect on CSF-1 serum concentration, which was still increased 19 days after injection of 50 mg/kg. This result may explain the high dose of AFS98 (50 mg/kg) required to achieve a therapeutic effect, since the mAb must compete with CSF-1 for CD115 binding.

### Anti-mouse CD115 mAb Treatment Delays the Appearance of Mammary Tumors in PyMT Mice

**Figure 3:**
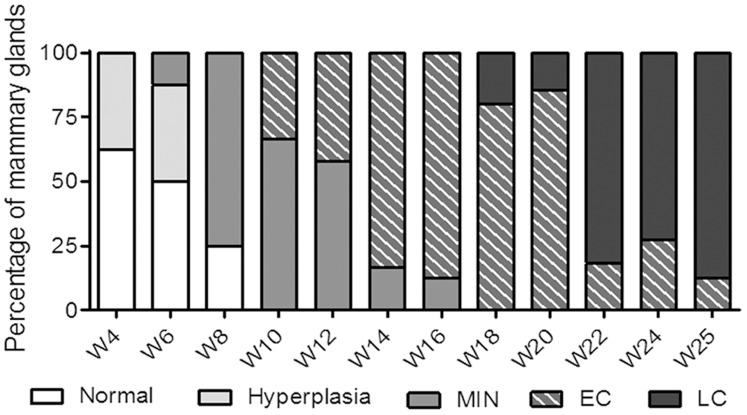
Progression of mammary tumors in C57BL/6 PyMT mice. Percentile distribution of tumor stages characterized by histopathology. Four to six mammary glands were removed from 2 to 6 PyMT mice at the indicated ages.

Transgenic MMTV-PyMT mice, expressing the polyoma middle T oncogene under control of the mouse mammary tumor virus promoter, spontaneously develop mammary tumors with stages comparable to the human situation [16], [39]. Based on the four-stage classification described in PyMT mice bred on a C3H/B6 background by Lin et al [39], we have characterized histologically the progression of mammary tumors in PyMT mice on the C57BL/6 genetic background (**Figure 3**). At 4 weeks of age, a few mammary glands were hyperplasic with epithelial cells proliferating within acini and forming focal or multifocal clusters on terminal ducts. In addition to the hyperplasia, adenomas (mammary intraepithelial neoplasia MIN) were observed at 6 weeks of age, characterized by solid sheets of proliferating epithelial cells completely filling the acini but still confined by a basement membrane. Foci of leukocytic infiltration were associated with increased vascularization. The initial stage of malignant transition, termed early carcinoma (EC), was first detected starting at 10 weeks of age. EC was characterized by a change in nuclear morphology, the disappearance of basement membranes and high density leukocyte infiltrates surrounding the acini. Mammary tumors became palpable between 13 and 14 weeks of age and on week 16, all mice had palpable tumors. Advanced invasive (late) carcinomas (LC) appeared around 18 weeks of age, characterized by solid sheets of epithelial cells and the loss of acinar structures, with the disappearance of basement membranes and the presence of necrotic areas. LC were always associated with lung metastasis.

**Figure 4:**
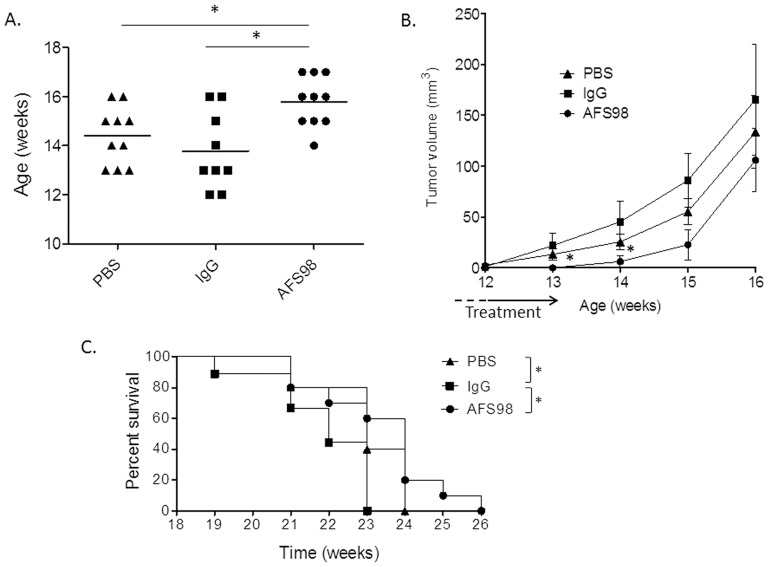
Appearance and progression of palpable mammary tumors are delayed in PyMT mice treated with AFS98. At 10 weeks of age, MMTV-PyMT mice were treated with 50 mg/kg of mAb AFS98, control rat IgG or PBS, injected IP 3 times per week from week 10 to week 13. **A**. The appearance of palpable tumors was delayed in AFS98-treated mice. The mean age (in weeks ± SEM) of palpable tumor detection is shown for each of the 3 mouse groups. * Mann-Whitney’s test p<0.05 compared with Rat IgG and PBS-treated mouse group (n = 10). **B**. Tumor volumes are shown as means ± SEM. * significant difference in tumor volume using Mann-Whitney’s test, p<0.05 between groups treated with Rat IgG and PBS (n = 15). **C**. Percentages of surviving mice in each group. * Log-rank test p<0.05 compared with the indicated mouse group (n = 10).

We aimed at studying the effect of anti-CD115 mAb treatment on the development of these mammary tumors. On tumor sections at the EC stage, all F4/80-positive infiltrating macrophages were stained with an anti-CD115 antibody (**Figure S3**). In contrast, PyMT tumor cells do not express CD115 [16] and therefore cannot be directly targeted by AFS98 treatment. The mAb was administered (50 mg/kg, IP 3 times per week) to PyMT mice from week 10 to 13, at the time of MIN to EC transition (**Figure 3**). Palpable mammary tumors appeared significantly later (15.8+/−0.9 weeks, n = 10) than in mice treated with isotype control (13.8+/−1.4 weeks, p = 0.0112, n = 10) or with PBS (14.4+/−1.1 weeks, p = 0.020, n = 10) (**Figure 4A**). Tumor sizes were significantly reduced with AFS98 at week 13 (p = 0.0149 vs PBS and p = 0.0098 vs rat IgG) and at week 14, after termination of the treatment (p = 0.0183 vs rat IgG- and p = 0.0212 vs PBS-treated mice) (**Figure 4B**). AFS98 treatment significantly prolonged survival when compared with rat IgG (p = 0.018) (**Figure 4C**). Of note, treatment with rat IgG had a deleterious effect on survival when compared to the PBS-treated group (p = 0.0283). The therapeutic effect of the anti-CD115 mAb was lost two weeks after cessation of the treatment. This may be explained by the dramatic upregulation of circulating CSF-1, occurring in C57BL/6 (Figure S2) and in PyMT mice (data not shown) following multiple mAb administration.

**Figure 5:**
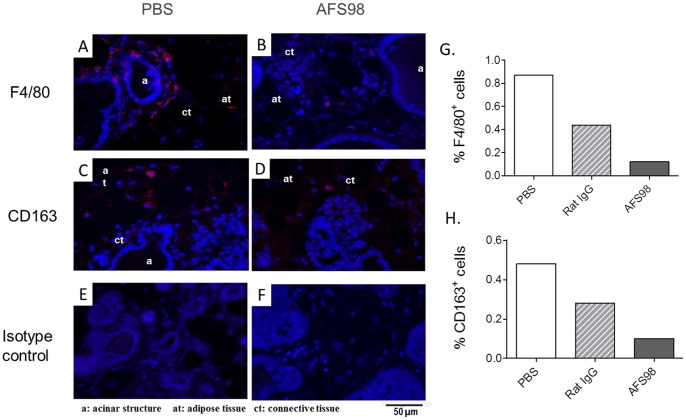
Anti-CD115 mAb treatment depletes TAMs and M2-type macrophages in PyMT mouse tumors. F4/80 (A, B) or CD163 (C, D) expression in mammary tumors sampled at week 14 from mice treated with either PBS (A, C) or mAb AFS98 (B, D). Staining with irrelevant isotype control is shown in E, F. Sections shown were obtained from one mouse representative of 3 analyzed per group. Blue: DNA; Red: F4/80^+^ (A, B) or CD163^+^ (C, D) macrophages. F4/80 (G) and CD163 (H) staining were quantified in the tumors from mice treated with either PBS, control rat IgG or mAb AFS98.

As found in the EL4 SC tumor model, the macrophage marker F4/80 was strongly decreased in primary mammary tumors from PyMT mice treated with 50 mg/kg AFS98 sampled one week after cessation of the treatment, compared to PBS-treated mice (**Figure 5A, B, G**). The number of M2-polarized macrophages identified by CD163 staining was also reduced in tumors from anti-CD115-treated mice (**Figure 5C, D, H**). However, angiogenesis was not detectably inhibited as found by CD31 staining of these tumors, sampled one week after cessation of the treatment (data not shown).

### Late Anti-mouse CD115 mAb Treatment Prolongs the Survival of PyMT Mice and Potentiates the Effect of Chemotherapy

**Figure 6:**
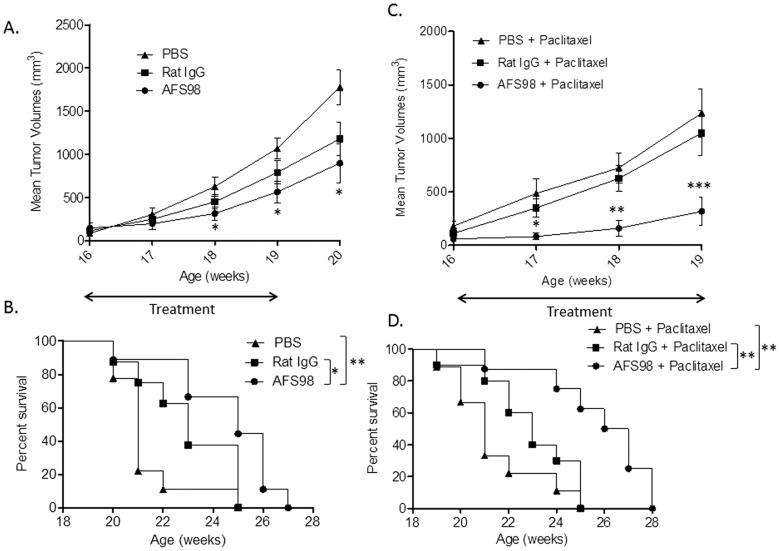
Therapeutic effects of anti-mouse CD115 mAb alone or combined with Paclitaxel in the PyMT mouse model. At 16 weeks of age, transgenic PyMT mice were administered 50 mg/kg of mAb AFS98 or isotype control or PBS, IP, 3 times per week for 4 weeks, alone (A, B) or combined (C, D) with Paclitaxel at 5 mg/kg, IP, once a week for 3 weeks. **A.** Tumor volumes are represented as means ± SEM. * Mann-Whitney’s test p<0.05 for AFS98- compared to PBS-treated mice. At week 20, * p<0.05 for AFS98- and Rat IgG- vs PBS-treated mice. **B.** The percentage of surviving mice was significantly increased in the AFS98-treated group. * Log-rank test p<0.05 between AFS98- and Rat IgG-treated groups and ** p<0.005 between AFS98- and PBS-treated mice (n = 10). **C.** Tumor volumes are represented as means ± SEM. * Mann-Whitney’s test p<0.05 for the combination Paclitaxel+AFS98 compared with Paclitaxel+PBS, and p<0.01 compared with Paclitaxel+Rat IgG. **p<0.01 and ***p<0.001 for Paclitaxel+AFS98 compared with Paclitaxel+Rat IgG or PBS (n = 10). **D.** The percentage of surviving mice was significantly increased by AFS98 combined with Paclitaxel. ** Log-rank test p<0.01 for mice treated with Paclitaxel+AFS98 compared to Paclitaxel+Rat IgG or PBS.

We then studied the effect of anti-CD115 mAb treatment administered later during tumor progression. AFS98 was dosed (50 mg/kg, IP 3 times per week, for 3 weeks) starting from week 16, when all tumors were palpable, mostly at the EC stage without yet reaching the LC stage **(** **Figure 3** **)**. One week after termination of the treatment (week 20), the sizes of primary tumors were significantly reduced in the AFS98-treated group compared to the PBS- (p = 0.0101, n = 10) but not to the IgG-treated mice (p = 0.1457, n = 10) (**Figure 6A**). Survival was significantly prolonged by AFS98 compared to either PBS (p = 0.0031) or IgG (p = 0.0474) (**Figure 6B**). As seen when treatment was initiated earlier (**Figure 5**), depletion of CD163^+^ macrophages was observed in tumors from mice treated with AFS98 (**Figure S4**).

To study the effect of the anti-CD115 mAb in association with chemotherapy, we combined the latter AFS98 treatment with Paclitaxel (5 mg/kg, IP 1 time per week). Paclitaxel at this dose did not show significant therapeutic effects, but a trend towards reduction in tumor growth. In PyMT mice treated with Paclitaxel combined with AFS98, the sizes of primary tumors were significantly reduced compared with PBS (p = 0.0155, p = 0.0039 and p = 0.0009 at week 17, 18 and 19, respectively, n = 10) or rat IgG (p = 0.0077, p = 0.0022 and p = 0.0005 at week 17, 18 and 19, respectively, n = 10) (**Figure 6C**). Survival was significantly prolonged when Paclitaxel was combined with AFS98, compared to either PBS (p = 0.0014) or IgG (p = 0.00072) (**Figure 6D**), showing that anti-CD115 mAb treatment could potentiate the effect of chemotherapy.

### Anti-mouse CD115 mAb Inhibits Osteoclasts and Bone Destruction in a Breast Cancer Bone Metastasis Model

**Figure 7:**
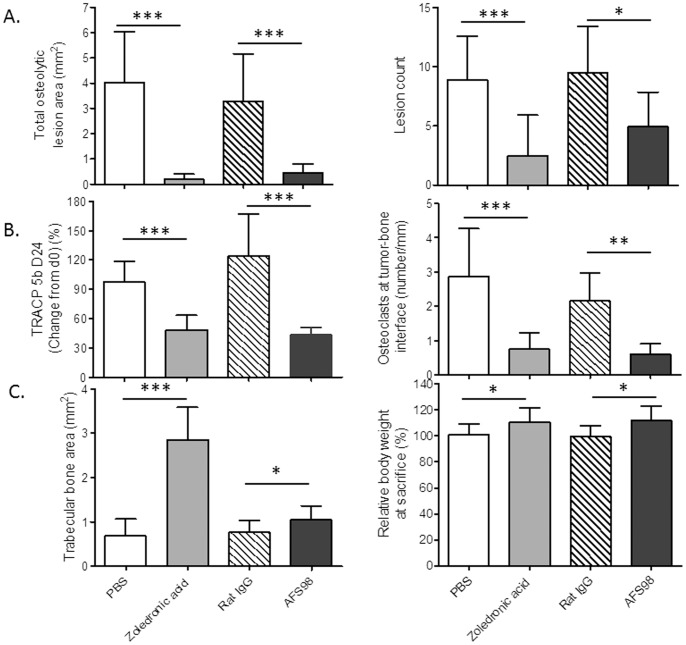
Therapeutic and osteoclast-inhibitory effects of anti-mouse CD115 mAb in a breast cancer metastasis-induced osteolysis model. Athymic nude mice implanted with MDA-MB231 tumor cells were treated with mAb AFS98, control rat IgG (both IP at 50 mg/kg, IP 3 times a week), zoledronic acid (0.1 mg/kg) or PBS (both SC, on days 0 and 14). 7.**A.** Bone-protective effect of anti-CD115 mAb. Osteolytic lesions were measured at the end of the experiment. *Left*: Total osteolytic lesion areas (mm^2^, mean+SD) are shown as the sums of the areas of the bone lesions in right and left tibia and femur. *Right*: Number of osteolytic lesions (mean+SD). The results are shown as the counts of individual bone lesions in right and left tibia and femur. 7.**B.** Osteoclast-inhibitory effect of anti-CD115 mAb. *Left*: The osteoclast marker TRACP 5b was titrated in serum sampled at day 24. Results are expressed as relative change (%) in serum TRACP 5b from day 0 to day 24 (mean+SD). *Right*: Sections from hind limbs of each animal were stained for TRACP 5b and osteoclast numbered at the tumor-bone interface (mean+SD). Osteoclast numbers were divided by the length of this interface. 7.**C.** Effects of anti-CD115 mAb on trabecular bone and inhibition of cancer-induced weight loss. *Left*: Trabecular bone area (mm^2^, mean+SD) of each animal was determined by histomorphometry. The results shown are the sums of trabecular bone areas in right and left tibia and femur. *Right*: Relative body weight at sacrifice (%, relative to day 0, mean+SD). *p<0.05; **p<0.01 and ***p<0.001 compared with control, using the statistical tests indicated in Materials and Methods.

We investigated the effect of CD115 blockade on metastasis-induced osteoclastic bone degradation. To this end, we made use of a model in which human breast cancer cells are injected in the left cardiac ventricles of athymic mice to induce osteolytic bone metastasis [37], [41]. MDA-MB231 cells are known to secrete human CSF-1 [42], which can activate murine CD115 [7]. MDA-MB231 cells are reported to also express human CD115 [42], but mAb AFS98 is specific for murine CD115 [34]; therefore, in this model, anti-CD115 mAb treatment will only target the murine myeloid cell compartment. Treatment of mice with 50 mg/kg mAb AFS98 potently inhibited the development of the osteolytic lesions visualized by X-ray radiography (**Figure 7A**). AFS98 displayed an activity similar to zoledronic acid, a standart-of-care inhibitor of osteolysis which was used as positive control. Both compounds diminished the area as well as the number of osteoclastic lesions, compared with PBS or rat IgG controls. AFS98 and zoledronic acid were equally potent at decreasing osteoclast numbers, as shown by the osteoclast marker Tartrate-resistant acid phosphatase 5b (TRACP 5b) which was diminished in the serum of mice at days 17 (data not shown) and 24 (**Figure 7B**). As measured by histomorphometry, osteoclast numbers at the tumor-bone interface were strongly decreased by AFS98 or zoledronic acid. Both compounds increased total and trabecular bone areas, with a stronger effect of zoledronic acid on trabecular bone areas (**Figure 7C**). Cancer-associated weight loss was inhibited similarly by zoledronic acid or AFS98. The MDA-MB231 cell line used in this study stably expresses GFP [37]. Whole body tumor burden analyzed by fluorescence imaging was not modified by either anti-CD115 mAb or zoledronic acid treatment, compared with control animals (data not shown). Altogether, these results show that the anti-CD115 mAb inhibits metastasis-induced bone destruction by targeting osteoclasts.

## Discussion

This study demonstrates for the first time that targeting CD115 on cancer host cells using a blocking mAb (i) depletes CD163^+^ M2-type TAMs and inhibits tumor growth in either ectopic or spontaneous cancer models; (ii) has therapeutic activity at early or late cancer stages in a model recapitulating the progression of human breast cancer, where it potentiates the effect of chemotherapy; and (iii) inhibits metastasis-induced bone degradation by decreasing osteoclast numbers.

Attempts to target the CD115/CSF-1 pathway in order to inhibit TAMs in mouse tumor models have been reported using various strategies [5], [43]–[46] and notably using mAbs [47]–[50]. McDonald et al. found in two transplanted syngenic tumor models (AE5MG mesothelioma and LLC lung carcinoma) that an anti-mouse CD115 IgG_1_ named M279) depleted F4/80^+^/Gr1^−^ monocytes and TAMs but did not affect tumor growth [49]. Kubota et al, with the same mAb as the one used in this study, could inhibit the growth and metastasis of AX tumors and showed that TAMs were depleted [47]. In our study, treatment of EL4 tumors with mAb AFS98 resulted in slower tumor growth and in prolonged mouse survival. In agreement with the previous studies, the anti-CD115 mAb depleted tumor-associated F4/80^+^ cells. Our results show in addition that anti-CD115 mAb treatment inhibits the infiltration of CD163^+^ F4/80^+^ TAMs, corresponding to alternatively-activated or M2-type macrophages. This supports the role of M2-polarized TAMs in promoting tumor growth in the EL4 tumor model. We did not detect changes in tumor vasculature whether mice were treated or not with AFS98, in contrast to the results reported upon treatment of AX tumor-bearing mice [47]. This was unexpected, given the known role of macrophages in tumor angiogenesis [17]. The therapeutic effects observed in the EL4 tumor model may be related to the inhibition of other TAM functions, including immunomodulation.

Since a spontaneous tumor model would better reflect the natural process of cancer progression, we studied the effects of targeting CD115 with AFS98 in MMTV-PyMT mice. Lin *et al.* [16], [17] previously showed using this mouse model that the CD115/CSF-1 axis was involved in the progression of mammary cancer, regulated by macrophages. Our results show for the first time that treating PyMT mice with an anti-CD115 mAb around the time of malignant transformation delays the appearance of palpable primary tumors, suggesting that the CD115 axis is already involved in the early stages of carcinogenesis. Like in the EL4 cancer model, this therapeutic effect was correlated with the depletion of CD163^+^ M2-type TAMs. The fact that blocking CD115 gives a therapeutic benefit at this early timepoint during carcinogenesis suggests that preventing M2-type macrophage recruitment and/or of differentiation delays malignant transformation. Lin et al. [16] found that CSF-1 did not regulate the initial growth of primary PyMT tumors, but only their progression through its effect of TAMs and angiogenesis. Further studies are needed to determine whether the alternative CD115 ligand, IL-34, rather than CSF-1, might be involved in this early oncogenic process. Unexpectedly, we were unable to detect any inhibitory effect of the anti-CD115 mAb on tumor angiogenesis, despite the fact that macrophages - required for the “angiogenic switch” and malignant transformation [17] - were clearly diminished in the tumors sampled on week 14, one week after cessation of the treatment. Macrophage-induced angiogenesis might have occurred prior to initiation of the treatment, before progression from the neoplastic to the malignant stage around week 10. Indeed, Lin *et al.* found that macrophage infiltration preceeded PyMT tumor progression from the neoplastic to the early carcinoma stage [17]. However, we cannot rule out tumor angiogenesis was impacted only during the treatment period and was restored one week later, at the time of observation.

When administered to PyMT mice already bearing malignant tumors, the anti-CD115 mAb also inhibited tumor growth and prolonged mouse survival. Taken together with the previous results, this suggests that the CD115 pathway is involved both during early mammary tumorigenesis and at later stages of cancer progression. It had already been shown that progression through malignant and metastatic stages of PyMT tumors depended on CSF-1 and TAMs [5], [16]. This is further supported by the therapeutic effect of AFS98 observed in mice already bearing malignant tumors.

After only one administration of AFS98, serum CSF-1 concentrations were dramatically increased. This was expected because AFS98 blocks CSF-1 binding to CD115 and the cytokine is normally cleared from the circulation through receptor-mediated endocytosis. This strong effect on plasmatic CSF-1 levels has already been reported for another anti-CD115 mAb, M279 [49]. This phenomenon may represent a hurdle for anti-CD115 immunotherapy, since overwhelming cytokine activity after cessation of the treatment or in organs which are inaccessible to mAbs may result in counter-productive and even toxic effects. For administration to cancer patients, a mAb which could inhibit CD115 function without preventing CSF-1 capture and degradation may be preferable to a ligand-competitive mAb.

In an implanted PyMT tumor model, De Nardo *et al*. have recently shown that an anti-CSF-1 mAb could decrease TAM infiltration, but lacked therapeutic effect unless combined with the chemotherapeutic agent Paclitaxel [50]. In the PyMT spontaneous tumor model used in our study, the anti-CD115 mAb by itself could efficiently reduce tumor growth, suggesting that targeting the receptor might have a superior therapeutic effect than blocking CSF-1. The alternative ligand IL-34 could be responsible for activating the CD115 pathway within mammary tumors. De Nardo *et al.* found that cytotoxic therapy induced both CSF-1 and IL-34 production by mammary carcinoma cells, increasing TAM infiltration. In mice bearing xenografted MCF-7 breast tumors, a PEG-conjugated anti-CSF-1 Fab was reported to have therapeutic effect, reducing macrophage recruitment to the tumor and reversing tumor resistance to combined chemotherapy [48]. In line with these previous studies, our results in the PyMT mouse model show that anti-CD115 mAb potentiates the effect of Paclitaxel for inhibiting tumor growth and prolonging survival, providing another piece of evidence that targeting TAMs will ameliorate the efficacy of chemotherapeutic treatments.

The inhibition of metastasis-induced osteolysis by anti-CD115 immunotherapy is demonstrated for the first time in this study. The breast cancer metastasis model addresses the effect of the CD115 mAb on bone-resorbing osteoclasts induced by tumor cells secreting CSF-1. Since mAb AFS98 does not bind human CD115, it could not directly affect the growth of MDA-MB231 tumor cells. Small molecule TK inhibitors targeting CD115 have been shown efficacious in inhibiting cancer-induced osteolysis [46], [51]–[53], although it remained unclear whether this was due to an effect on tumor cells, on mouse osteoclasts, or even on another TKR - since small molecule TK inhibitors generally lack selectivity. Our results show for the first time that a mAb targeting host CD115^+^ myeloid cells can inhibit bone degradation induced by breast cancer metastases. AFS98 was as potent as a biphosphonate in decreasing osteoclast numbers and osteolytic lesions. Importantly, the mAb also prevented cancer-induced weight loss, suggesting that anti-CD115 immunotherapy could improve the quality of life for cancer patients.

Together, these results show that targeting CD115^+^ cells in the tumor microenvironment with a function-blocking mAb can have therapeutic effects through the dual inhibition of TAMs and osteoclasts. They support the clinical development of an anti-human CD115 mAb, which might be more specific and less toxic than a TKI, in cancer indications characterized by TAM infiltration and osteolytic bone metastases.

## Supporting Information

Figure S1 **Competition of AFS98 with mCSF-1 or hCSF-1 for mouse CD115 binding.** Microplate wells were coated with 0.1 µg of either mouse or human CSF-1 and the binding of mouse M-CSFR-Fc added at 0.1 µg/mL was assessed in the presence of 4 concentrations of AFS98 or control mAb (Rat IgG_2a_). Binding of mouse CD115-Fc was detected using a HRP conjugated antibody that recognized the Fc part of the recombinant antigen. MAb AFS98 dose-dependently blocks mCSF-1 and hCSF-1 binding to mCD115.
(DOCX)

Figure S2 **Treatment with mAb AFS98 strongly increases serum CSF-1.** C57BL/6 mice were injected IP with mAb AFS98 at 10, 25 or 50 mg/kg or PBS. Serum was collected at the indicated days after injection and titrated for mCSF-1 by ELISA (Duoset, R&D Systems).
(DOCX)

Figure S3 **Double immunofluorescence staining for F4/80 and CD115 on FFPE sections from MMTV-PyMT mouse mammary tumors.** To better characterize the CD115-positive cells in mammary tumors from MMTV-PyMT mice, Formalin-Fixed Paraffin-Embedded sections from tumors at the EC stage were double-stained with antibodies to F4/80 and CD115 (rabbit anti-CD115, C20, Santa Cruz). Not all CD115-positive cells co-expressed F4/80, but all F4/80-positive macrophages were also stained with the anti-CD115 antibody, reflecting a tumor-associated macrophage phenotype.
(DOCX)

Figure S4 **TAM and M2-type macrophage inhibitory effects of anti-CD115 mAb administered to PyMT mice starting at 16 weeks of age.** F4/80 (A, B) or CD163 (C, D) expression in mammary tumors sampled at week 20 from mice treated with either PBS (A, C) or mAb AFS98 (B, D). Staining with irrelevant isotype control is shown in E, F. Sections shown were obtained from one mouse representative of 3 analyzed per group. Blue: DNA; Red: F4/80^+^ (A, B) or CD163^+^ (C, D) macrophages. Sections shown were obtained from one tumor representative of 3 analyzed per group. F4/80 (G) and CD163 (H) staining were quantified in the tumors shown in A-D.
(DOCX)
