# Supplementary material for: Therapeutic Effects of Anti-CD115 Monoclonal Antibody in Mouse Cancer Models through Dual Inhibition of Tumor-Associated Macrophages and Osteoclasts
Source: PLoS One. 2013 Sep 3;8(9):e73310. doi: 10.1371/journal.pone.0073310 (PMC3760897; doi:10.1371/journal.pone.0073310)
Supplement: Figure S1 — Competition of AFS98 with mCSF-1 or hCSF-1 for mouse CD115 binding. Microplate wells were coated with 0.1 µg of either mouse or human CSF-1 and the binding of mouse M-CSFR-Fc added at 0.1 µg/mL was assessed in the presence of 4 concentrations of AFS98 or control mAb (Rat IgG2a). Binding of mouse CD115-Fc was detected using a HRP conjugated antibody that recognized the Fc part of the recombinant antigen. MAb AFS98 dose-dependently blocks mCSF-1 and hCSF-1 binding to mCD115. (DOCX) [file pone.0073310.s001.docx]

**Figure S1**

**Competition of AFS98 with mCSF-1 or hCSF-1 for mouse CD115 binding.** Microplate wells were coated with 0.1 µg of either mouse or human CSF-1 and the binding of mouse M-CSFR-Fc added at 0.1 µg/mL was assessed in the presence of 4 concentrations of AFS98 or control mAb (Rat IgG_2a_). Binding of mouse CD115-Fc was detected using a HRP conjugated antibody that recognized the Fc part of the recombinant antigen. MAb AFS98 dose-dependently blocks mCSF-1 and hCSF-1 binding to mCD115.
