# Supplementary material for: Therapeutic Effects of Anti-CD115 Monoclonal Antibody in Mouse Cancer Models through Dual Inhibition of Tumor-Associated Macrophages and Osteoclasts
Source: PLoS One. 2013 Sep 3;8(9):e73310. doi: 10.1371/journal.pone.0073310 (PMC3760897; doi:10.1371/journal.pone.0073310)
Supplement: Figure S2 — Treatment with mAb AFS98 strongly increases serum CSF-1. C57BL/6 mice were injected IP with mAb AFS98 at 10, 25 or 50 mg/kg or PBS. Serum was collected at the indicated days after injection and titrated for mCSF-1 by ELISA (Duoset, R&D Systems). (DOCX) [file pone.0073310.s002.docx]

**Figure S2**

**Treatment with mAb AFS98 strongly increases serum CSF-1.** C57BL/6 mice were injected IP with mAb AFS98 at 10, 25 or 50 mg/kg or PBS. Serum was collected at the indicated days after injection and titrated for mCSF-1 by ELISA (Duoset, R&D Systems).
