# Supplementary material for: Therapeutic Effects of Anti-CD115 Monoclonal Antibody in Mouse Cancer Models through Dual Inhibition of Tumor-Associated Macrophages and Osteoclasts
Source: PLoS One. 2013 Sep 3;8(9):e73310. doi: 10.1371/journal.pone.0073310 (PMC3760897; doi:10.1371/journal.pone.0073310)
Supplement: Figure S3 — Double immunofluorescence staining for F4/80 and CD115 on FFPE sections from MMTV-PyMT mouse mammary tumors. To better characterize the CD115-positive cells in mammary tumors from MMTV-PyMT mice, Formalin-Fixed Paraffin-Embedded sections from tumors at the EC stage were double-stained with antibodies to F4/80 and CD115 (rabbit anti-CD115, C20, Santa Cruz). Not all CD115-positive cells co-expressed F4/80, but all F4/80-positive macrophages were also stained with the anti-CD115 antibody, reflecting a tumor-associated macrophage phenotype. (DOCX) [file pone.0073310.s003.docx]

**Figure S3**

**Double immunofluorescence staining for F4/80 and CD115 on FFPE sections from MMTV-PyMT mouse mammary tumors.** To better characterize the CD115-positive cells in mammary tumors from MMTV-PyMT mice, Formalin-Fixed Paraffin-Embedded sections from tumors at the EC stage were double-stained with antibodies to F4/80 and CD115 (rabbit anti-CD115, C20, Santa Cruz). Not all CD115-positive cells co-expressed F4/80, but all F4/80-positive macrophages were also stained with the anti-CD115 antibody, reflecting a tumor-associated macrophage phenotype.

**
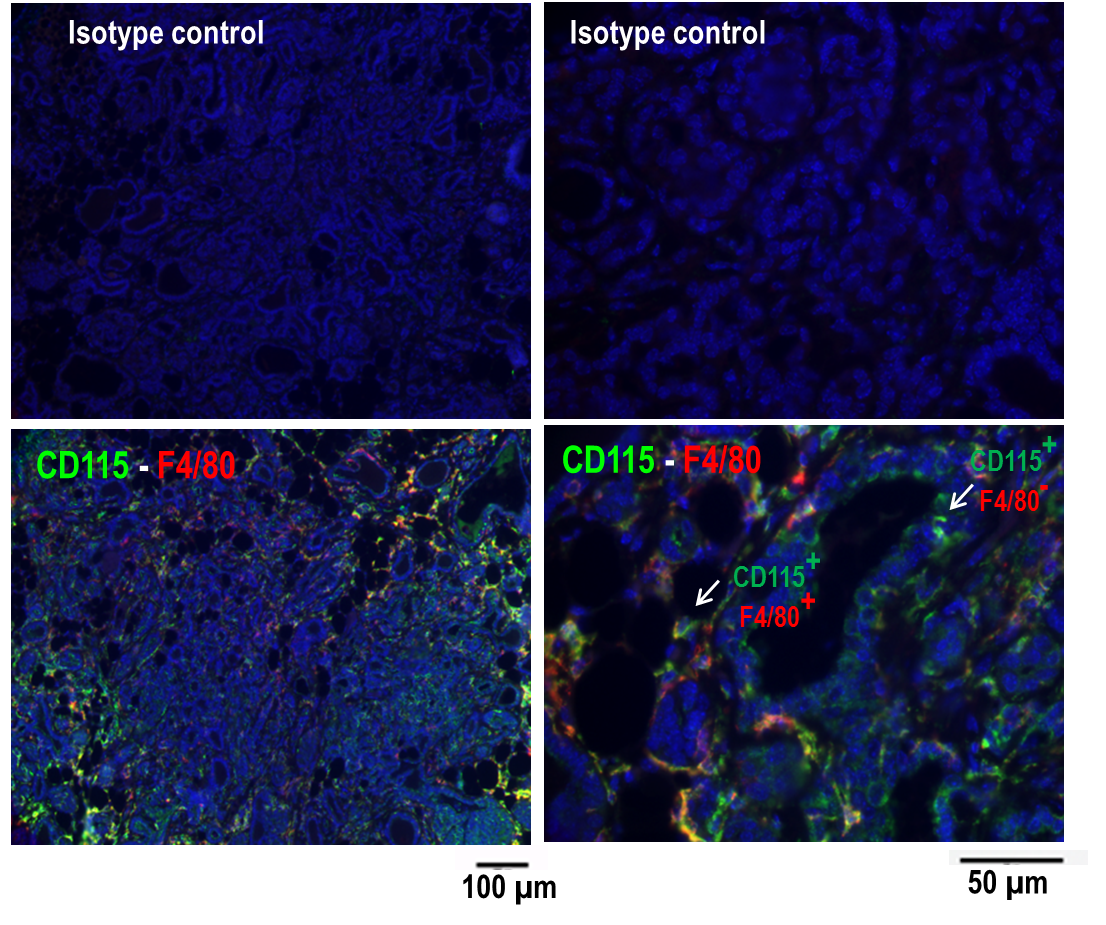
**
