# Supplementary material for: Therapeutic Effects of Anti-CD115 Monoclonal Antibody in Mouse Cancer Models through Dual Inhibition of Tumor-Associated Macrophages and Osteoclasts
Source: PLoS One. 2013 Sep 3;8(9):e73310. doi: 10.1371/journal.pone.0073310 (PMC3760897; doi:10.1371/journal.pone.0073310)
Supplement: Figure S4 — TAM and M2-type macrophage inhibitory effects of anti-CD115 mAb administered to PyMT mice starting at 16 weeks of age. F4/80 (A, B) or CD163 (C, D) expression in mammary tumors sampled at week 20 from mice treated with either PBS (A, C) or mAb AFS98 (B, D). Staining with irrelevant isotype control is shown in E, F. Sections shown were obtained from one mouse representative of 3 analyzed per group. Blue: DNA; Red: F4/80+ (A, B) or CD163+ (C, D) macrophages. Sections shown were obtained from one tumor representative of 3 analyzed per group. F4/80 (G) and CD163 (H) staining were quantified in the tumors shown in A-D. (DOCX) [file pone.0073310.s004.docx]

**Figure S4**

**TAM and M2-type macrophage inhibitory effects of anti-CD115 mAb administered to PyMT mice starting at 16 weeks of age.** F4/80 (A, B) or CD163 (C, D) expression in mammary tumors sampled at week 20 from mice treated with either PBS (A, C) or mAb AFS98 (B, D). Staining with irrelevant isotype control is shown in E, F. Sections shown were obtained from one mouse representative of 3 analyzed per group. Blue: DNA; Red: F4/80^+^ (A, B) or CD163^+^ (C, D) macrophages. Sections shown were obtained from one tumor representative of 3 analyzed per group. F4/80 (G) and CD163 (H) staining were quantified in the tumors shown in A-D.


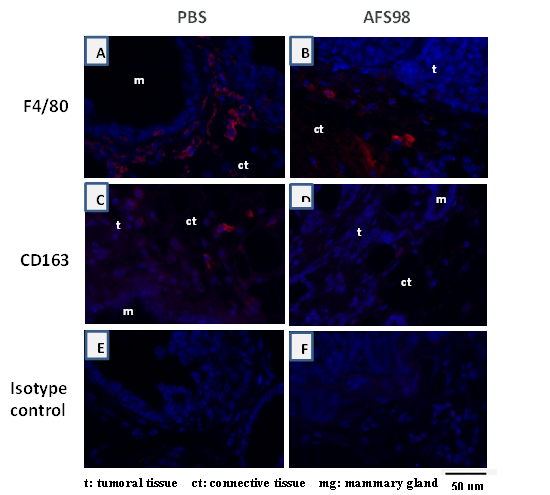


**

H.

G.
